# A lightweight perceptual-guided VQVAE for high-fidelity image compression

**DOI:** 10.1038/s41598-026-53855-z

**Published:** 2026-05-19

**Authors:** Zhisong Bie, Yunyang Kuang, Haobo Lei, Hongxia Bie

**Affiliations:** School of Artificial Intelligence, Beijing University of Posts and Telecommunications, Beijing, 100876 China

**Keywords:** Lightweight, Low-bit-rate, SR-Guided codec, Multi-level loss, Engineering, Mathematics and computing

## Abstract

Low-bit-rate image compression faces a persistent quality-efficiency dilemma: lightweight models such as VQ-VAE produce perceptually degraded reconstructions, while high-quality alternatives like VQGAN and diffusion models incur prohibitive computational costs. To bridge this gap, we propose HiRes-VQ, a lightweight perceptual-guided VQ-VAE that achieves high-fidelity reconstruction without sacrificing efficiency. Built upon VQ-VAE-2’s hierarchical quantization, HiRes-VQ introduces two key innovations: (1) an asymmetric encoder-decoder architecture, where the encoder hierarchically extracts semantic features at multiple spatial scales and the decoder reconstructs low-frequency structures and high-frequency textures through separate frequency-domain pathways, together ensuring pixel-level fidelity; and (2) a multi-scale perceptual alignment loss that jointly optimizes pixel accuracy, semantic feature consistency, and style statistics, enabling perceptual-quality gains without compromising structural metrics. With only 3.21M parameters, HiRes-VQ achieves 18%–40% fidelity gains over similar-sized baselines on FFHQ-256 and ImageNet-256 across both pixel-level and semantic-level metrics, while surpassing high-complexity models such as VQGAN and OptVQ in quality-efficiency trade-off. Ablation experiments confirm that the dual-path decoder and the perceptual loss serve complementary roles, together enabling significant improvements in both pixel-level fidelity and semantic perceptual quality. These results demonstrate that HiRes-VQ effectively resolves the quality-efficiency dilemma, offering a practical solution for resource-constrained deployment.

## Introduction

With the rapid advancement of digital multimedia technology, image and video data have experienced exponential growth. Efficient data compression and reconstruction techniques have thus become a key research focus in both academia and industry. Traditional image compression methods, such as JPEG^1^ and HEVC^2^, can achieve high compression ratios. However, they often introduce noticeable block artifacts and blurring at low bit rates, which significantly degrades visual quality. In recent years, deep learning-based generative models^3^ have made significant progress in the field of image compression and reconstruction. By learning the intrinsic priors of images in a data-driven manner, they enable higher-quality reconstruction even under low-bit-rate conditions.

Among various generative models, Vector Quantized Variational Autoencoders (VQ-VAE)^4^ and their subsequent improvements^5–9^, such as VQ-VAE-2^5^, have enabled lightweight image compression and reconstruction through the introduction of vector quantization. However, VQ-VAE typically employs a Simple Mean Squared Error (MSE) as the reconstruction loss and utilizes a relatively basic codec architecture. These limitations cause the reconstructed images to lose significant detail and semantic features, manifesting as issues like image blurring and texture distortion. This results in a clear deficit in reconstruction quality. To enhance reconstruction quality, subsequent research has proposed more complex generative models, such as VQGAN^10^ and Diffusion Models^11–15^. While these methods achieve improved quality, they also lead to a sharp increase in model complexity. They fail to achieve a satisfactory balance between computational resource consumption and reconstruction quality. Consequently, their application in resource-constrained scenarios—such as on mobile devices or in real-time communication—remains limited.

Currently, generative models for compression-reconstruction tasks commonly face a ”quality-efficiency dilemma”. On one hand, lightweight models (e.g. VQ-VAE) suffer from low reconstruction quality and struggle to meet high-fidelity requirements. On the other hand, models capable of high-quality reconstruction (e.g. VQGAN, diffusion models) are often excessively complex and demand substantial computational resources. To address this dilemma, this paper aims to explore a novel model design that achieves a balance between lightweight architecture and high-quality reconstruction under low-bit-rate scenarios.

Specifically, we proposes HiRes-VQ, a lightweight yet high-quality image compression and reconstruction model based on an improved VQ-VAE framework. While maintaining the simplicity and efficiency of the VQ-VAE architecture, our work contributes in three key aspects:

- We propose a Super-Resolution Guided Codec (SR-Guided Codec) that achieves high-fidelity reconstruction at both pixel level and semantic level. By separately processing low-frequency and high-frequency information in its decoder, our method significantly enhances textural details and structural clarity while maintaining a lightweight model architecture.
- We design a multi-level loss function that integrates perceptual loss and style loss, utilizing high-level semantic features to guide model training. This achieves perceptual-level alignment of reconstruction quality with human visual perception, thereby enabling perceptual-guided optimization for enhanced perceptual fidelity.
- We conducted extensive subjective and objective evaluations on the FFHQ-256 and ImageNet-256 datasets. Our model achieves 18%−40% fidelity gains over similar-sized models on both datasets, with faster convergence and superior quality-efficiency trade-offs compared to larger SOTA models.

Through these improvements, our model achieves outstanding performance in low-bit-rate compression and reconstruction tasks, providing an effective solution to the quality-efficiency dilemma in practical applications.

**Fig. 1:**
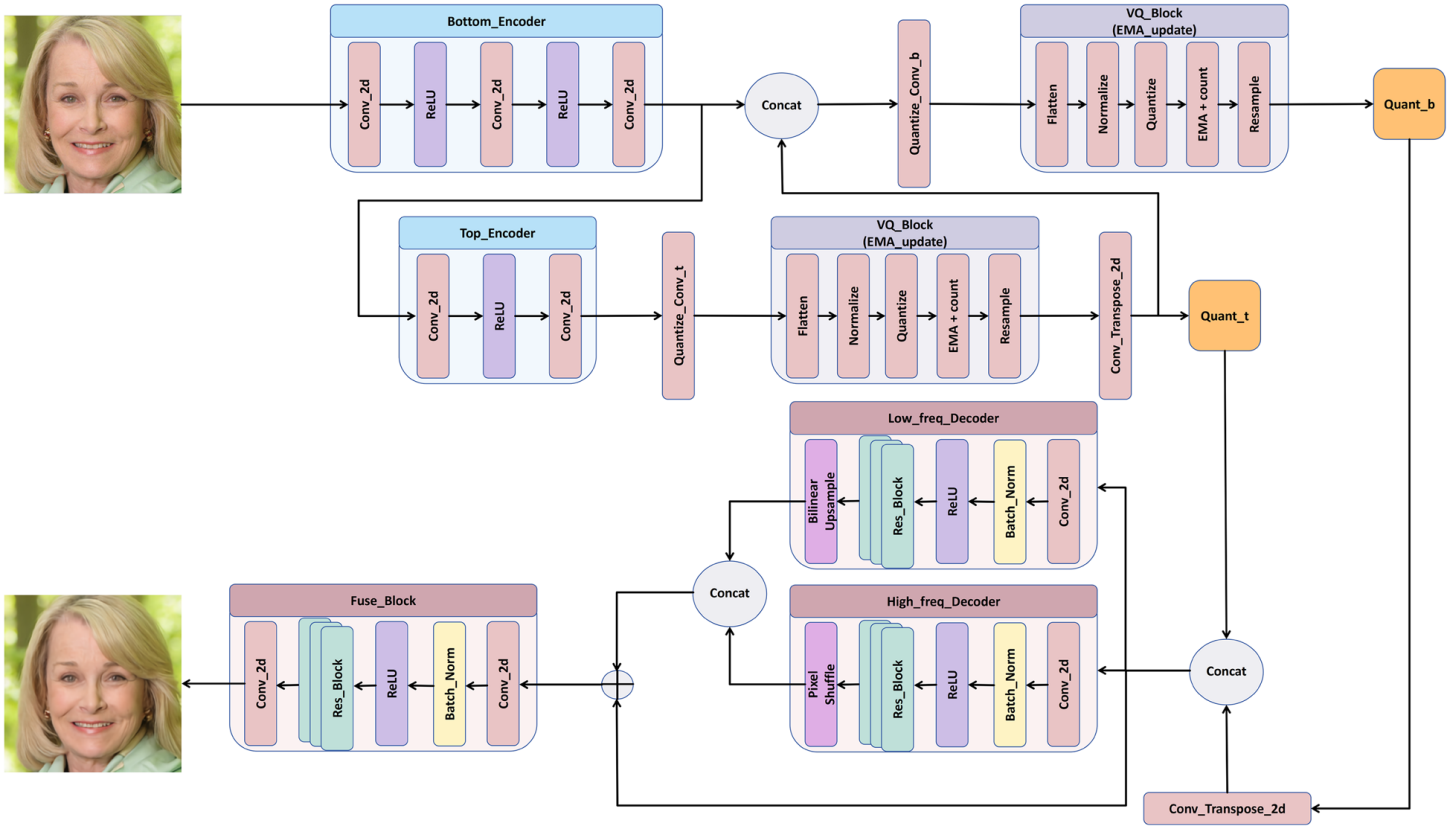
Overview of the proposed HiRes-VQ framework. Top: Hierarchical encoder and quantization modules, which compress the input image into a discrete latent representation via a two-level vector quantization process. Bottom: The core dual-path disentangled-fusion decoder, which reconstructs the image through a low-frequency path (for stable structure recovery) and a high-frequency path (for detailed texture reconstruction), followed by adaptive feature fusion.

## Related works

### Neural image compression

Image compression has been a fundamental research area for decades, evolving from hand-crafted standards to data-driven deep learning approaches. Traditional codecs such as JPEG^1^, JPEG 2000^16^, HEVC/H.265^2^ rely on manually designed transformation, quantization, and entropy coding pipelines optimized through extensive engineering. While these codecs achieve high compression ratios, they inevitably introduce visually objectionable artifacts like blocking, ringing, and blurring at low bit rates, as their rigid transformation bases and hand-tuned quantization tables cannot adapt to the vast diversity of image content. The emergence of deep learning has catalyzed a paradigm shift toward learned image compression^17–19^, where end-to-end neural networks jointly optimize the analysis transform, quantization, and synthesis transform. Ballé et al.^17^ pioneered end-to-end optimized compression using convolutional autoencoders with a differentiable entropy model, establishing a competitive baseline against JPEG 2000. Subsequent works incorporated hyperpriors^18^ and autoregressive context models^19^ to further capture spatial dependencies in the latent representation, progressively closing the gap with state-of-the-art codecs. However, these methods primarily optimize for pixel-level distortion metrics (MSE/MS-SSIM) and tend to produce overly smooth reconstructions lacking realistic textural details, motivating the exploration of generative approaches that prioritize perceptual quality.

### Vector quantized generative models

Vector Quantized Variational Autoencoder (VQ-VAE)^4^ introduced a discrete latent space for generative modeling by replacing the continuous latent variables of standard VAEs with a learned codebook of embedding vectors. This discrete representation naturally lends itself to compression, as the latent codes can be efficiently entropy-coded. VQ-VAE-2^5^ extended this framework with a hierarchical multi-scale quantization architecture: a bottom-level quantizer captures local textures and fine-grained features, while a top-level quantizer models global structure and long-range dependencies. This hierarchical design significantly improves generation fidelity and has become a backbone for VQ-based generative models.

Since these seminal works, substantial efforts have been devoted to improving VQ-based representations along several axes. On the quantization front, RQ-VAE^7^ recursively quantizes the residual between the original feature and its quantized counterpart, enabling finer approximation with a stack of shallow codebooks. SQ-VAE^6^ and HQ-VAE^8^ reformulate vector quantization within a variational Bayes framework with stochastic quantization and self-annealing, addressing training instability and codebook collapse. FSQ^20^ demonstrates that a simple finite scalar quantization scheme, without the complex commitment losses or codebook update mechanisms of traditional VQ, can match or even surpass VQ-VAE performance. On the training stability front, improved codebook update strategies^21^ using straight-through gradient estimation and periodic codebook reset have been shown to yield higher codebook utilization and more effective latent representations. OptVQ^22^ addresses local optima in codebook learning through an optimal transport formulation, achieving improved reconstruction quality at the cost of significantly increased model complexity.

### Generative adversarial and diffusion-based compression

To address the inherent blurriness of MSE-optimized reconstructions, researchers have integrated adversarial training into VQ frameworks. VQGAN^10^ augments VQ-VAE with a patch-based discriminator and perceptual loss, achieving dramatic improvements in perceptual quality—producing sharp, realistic textures even at high compression ratios. ViT-VQGAN^23^ further replaces convolutional encoders/decoders with vision transformers, leveraging self-attention to capture non-local dependencies. MoVQ^24^ introduces a spatially conditional normalization mechanism within the VQ decoder, enabling more accurate reconstruction of fine-grained details. Concurrently, diffusion models^11,15^ have emerged as a highly powerful class of generative models capable of producing images of unprecedented fidelity. By iteratively denoising a Gaussian prior conditioned on compressed representations, diffusion-based compression methods^9,12,13^ achieve remarkable perceptual quality. However, these quality improvements come at a steep cost: VQGAN variants typically employ hundreds of millions of parameters (e.g. 1.4B for VQGAN), and diffusion models require tens to hundreds of iterative inference steps, resulting in inference latency orders of magnitude higher than single-pass autoencoders. This renders them impractical for real-time or resource-constrained deployment scenarios.

### Lightweight architecture design and perceptual optimization

In parallel to the pursuit of quality, a substantial body of work has focused on model efficiency. Lightweight convolutional architectures such as MobileNet^25^ and EfficientNet^26^ demonstrate that depthwise separable convolutions, inverted residuals, and compound scaling can drastically reduce computational cost while preserving representational capacity. These design principles have been successfully adopted in learned compression^27^, where fast encoding/decoding is critical. Knowledge distillation, neural architecture search (NAS), and pruning have also been explored to reduce the parameter count and FLOPs of compression models. However, in the context of VQ-based generative compression, the benefits of these efficiency-oriented techniques have been underexplored; most existing lightweight VQ models sacrifice substantial reconstruction quality, particularly in high-frequency texture fidelity. Beyond compression, the broader field of image restoration faces analogous quality-efficiency trade-offs. For example, space-time distillation for video super-resolution^28^ leverages knowledge transfer from complex temporal models into efficient student networks to reduce inference cost while maintaining reconstruction accuracy. In event-based motion deblurring, learning dual-modality interactions between events and frames^29^ enables high-quality detail recovery through effective fusion of complementary sensor modalities. These works highlight a recurring theme—the need to maximize perceptual reconstruction quality under practical computational constraints—which equally motivates our approach in the context of learned image compression.

On the perceptual optimization front, Johnson et al.^30^ first demonstrated that feature-space distances computed by pre-trained deep networks (e.g. VGG) serve as effective training objectives for image transformation tasks, generating outputs that align more closely with human visual perception than pixel-wise losses. The Learned Perceptual Image Patch Similarity (LPIPS) metric^31^ further formalized this observation, providing a calibrated perceptual distance that has become a standard evaluation and training tool. Style loss^32^, formulated as the difference in Gram matrices of deep feature maps, captures texture statistics and global appearance, complementing perceptual losses by enforcing stylistic consistency. These perceptual objectives have been adopted in various generative compression pipelines^10,33,34^, but their integration has typically been within large, computationally expensive models. A systematic investigation of how to effectively embed multi-level perceptual supervision within a lightweight VQ-VAE architecture, to simultaneously achieve perceptual quality, training stability, and computational efficiency, remains absent from the literature.

### Summary and motivation

In summary, existing approaches to generative image compression populate a spectrum between two extremes: lightweight VQ-VAE variants that are efficient but produce blurry, perceptually degraded reconstructions, and high-complexity GAN or diffusion-based models that achieve impressive quality at the cost of massive parameter counts and prohibitive inference time. A critical gap exists for models that can deliver high perceptual fidelity within a tight parameter and computation budget suitable for edge and real-time deployment. Our proposed HiRes-VQ framework directly targets this gap through three key aspects: (1) an asymmetric encoder-decoder architecture, where the encoder hierarchically extracts image semantic features at multiple spatial scales, and the decoder simultaneously accounts for the reconstruction of both low- and high-frequency details in the frequency domain, thereby ensuring pixel-level fidelity; (2) a multi-level loss function that jointly imposes constraints on pixel-level and semantic-level fidelity, effectively improving semantic reconstruction quality without sacrificing pixel-level accuracy; and (3) retaining the lightweight nature of the VQ-VAE framework, directly addressing the quality-efficiency dilemma. This design philosophy distinguishes HiRes-VQ from both the lightweight baselines that optimize only for pixel-level accuracy and the high-complexity models that prioritize quality regardless of computational cost.

**Fig. 2:**
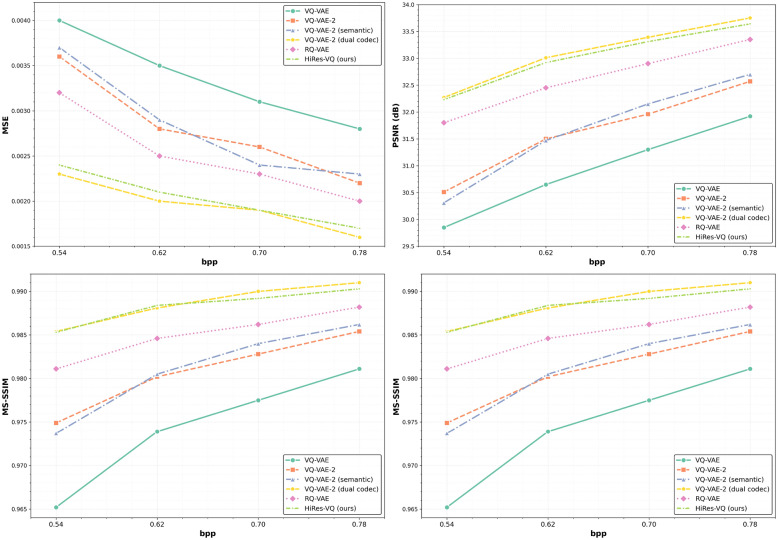
Pixel-level indexes on FFHQ-256.

**Fig. 3:**
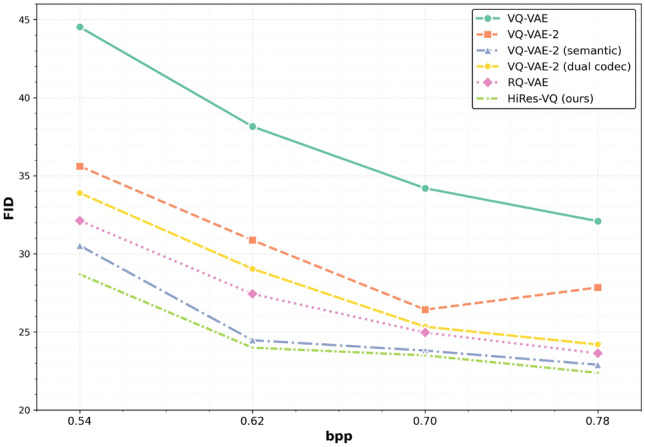
Semantic-level indexes on FFHQ-256.

## Methods

We propose HiRes-VQ, a perception-guided reconstruction framework designed for low-bit-rate scenarios (Fig. 1). By constructing an encoder-decoder architecture with multi-level semantic supervision and frequency-domain decoupling, and integrating a multi-head adaptive codebook optimization mechanism along with a multi-scale perceptual alignment loss, the framework achieves high-quality image reconstruction while maintaining a lightweight model structure.

### SR-guided codec

We propose a super-resolution guided codec (SR-Guided Codec) architecture that, while inheriting the hierarchical quantization framework of VQ-VAE-2, achieves a synergistic optimization of reconstruction quality and training efficiency by restructuring the internal information flow of the decoder and introducing a frequency-domain decoupling mechanism. The encoder employs a two-tier pyramid structure. The bottom encoder captures local textures and fine-grained semantic features of the image through downsampling convolutions. The top encoder performs a secondary downsampling on this basis to extract global semantic features. The features from both levels are then mapped into discrete codebook spaces via independent vector quantizers, forming an efficient hierarchical compressed representation.

The decoder section represents the core innovation of this architecture. We designed a Dual-path Disentangled-Fusion Decoder (DPDF-Decoder) to systematically address the inherent trade-off between reconstruction quality and training stability. This decoder employs a parallel dual-branch structure, with each branch dedicated to reconstructing features from distinct frequency domains:

1 $$\begin{aligned} \textbf{F}_{\text {low}} = \mathscr {U}_{\text {bilinear}}^{(2\times )}(\mathscr {U}_{\text {bilinear}}^{(2\times )}(\mathscr {C}(x))). \end{aligned}$$

- The low-frequency reconstruction branch employs a combination of progressive bilinear upsampling and lightweight convolutional modules, focusing on restoring the overall structure, smooth regions, and macroscopic contours of the image. By incorporating spatial-aware normalization layers and identity residual connections, this branch ensures stable gradient propagation through the deep network, effectively mitigating feature dispersion and mode collapse during training.

2 $$\begin{aligned} \textbf{F}_{\text {high}} = \mathscr {P}^{(2\times )}(\mathscr {P}^{(2\times )}(\mathscr {C}(x))). \end{aligned}$$

- The high-frequency reconstruction branch is built upon sub-pixel convolution (PixelShuffle) to form a detail-enhancement network, specifically designed to reconstruct edge textures, high-frequency details, and local abrupt features of the image. This branch employs channel rearrangement rather than interpolation operations to achieve resolution upscaling, fundamentally avoiding the checkerboard artifacts commonly induced by traditional transposed convolutions. Meanwhile, a compressed bottleneck layer design is adopted to maintain low computational overhead.

3 $$\begin{aligned} \textbf{F}_{\text {out}} = \mathscr {M}_{\text {fuse}}\left( \text {Concat}(\textbf{F}_{\text {low}}, \textbf{F}_{\text {high}}) \right). \end{aligned}$$

The outputs from the dual paths are integrated via an adaptive feature fusion module. This module dynamically adjusts the contribution weights of the two paths through a gating mechanism and leverages a multi-scale feature pyramid to achieve cross-resolution information complementarity.

### Multi-scale perceptual alignment loss function

We design a multi-scale perceptual alignment loss function aimed at collaboratively guiding the model optimization process from three dimensions: pixel-level, feature-level, and style statistics. This loss framework not only focuses on low-level pixel accuracy but also emphasizes the consistency of high-level semantic features. It thereby effectively preserves structural and textural semantic information that is crucial for visual perception during the compression and reconstruction process.

4 $$\begin{aligned} \begin{aligned} \mathscr {L}_{\text {total}} =&\lambda _{\text {mse}}\mathscr {L}_{\text {mse}} + \lambda _{\text {commit}}\mathscr {L}_{\text {commit}} + \lambda _{\text {perc}}\mathscr {L}_{\text {perc}} + \lambda _{\text {style}}\mathscr {L}_{\text {style}}. \end{aligned} \end{aligned}$$

The core loss function consists of four complementary components, forming progressive optimization objectives: the basic reconstruction loss ensures pixel-level fidelity; the quantization alignment loss maintains the effectiveness of the discrete representation space; the multi-scale perceptual loss guides semantic feature consistency; and the style statistical loss further reinforces the alignment of texture and global distribution. These loss terms are balanced through a dynamic weight scheduling mechanism, with varying emphasis during different training stages. Together, they drive the model toward convergence on the goal of high perceptual-quality reconstruction.

5 $$\begin{aligned} \mathscr {L}_{\text {mse}} = \mathbb {E}_{I \sim p_{\text {data}}} \left[ \Vert I_{\text {rec}} - I_{\text {gt}} \Vert _2^2 \right], \end{aligned}$$

6 $$\begin{aligned} \mathscr {L}_{\text {commit}} = \Vert \text {sg}[E(x)] - z_q \Vert _2^2 + \beta \Vert E(x) - \text {sg}[z_q] \Vert _2^2. \end{aligned}$$

The basic reconstruction loss uses Mean Squared Error (MSE) to measure pixel-level similarity, providing direct supervision for reconstruction. Additionally, we introduce a quantization alignment loss to regularize the discrete latent space, consisting of two parts: a commitment term that aligns encoder outputs with their nearest codebook vectors, and a codebook update mechanism that ensures effective feature representation. Together, these losses establish a stable optimization foundation.

7 $$\begin{aligned} \mathscr {L}_{\text {perc}} = \sum _{l \in \mathscr {L}} \alpha _l \cdot \frac{1}{N_l \cdot C_l \cdot H_l \cdot W_l} \sum _{i=1}^{N} \left\| \Phi _l(I_{\text {rec}}^{(i)}) - \Phi _l(I_{\text {gt}}^{(i)}) \right\| _1. \end{aligned}$$

To overcome the perceptual limitations of pixel-level similarity, we design a multi-scale perceptual alignment loss. This loss employs a pre-trained deep visual network (VGG-16) as a perceptual feature extractor. It performs matching across three distinct feature layers, targeting edge-texture preservation, local feature consistency, and high-level semantic concept alignment, respectively. This constraint guides the model to learn feature representations that are more sensitive to the human visual system.

8 $$\begin{aligned} \mathscr {L}_{\text {style}} = \sum _{l \in \mathscr {L}} \alpha _l \cdot \left\| G_l(I_{\text {rec}}) - G_l(I_{\text {gt}}) \right\| _1. \end{aligned}$$

Furthermore, we introduce a style statistical loss to enhance the texture realism and global coherence of the reconstructed images. This loss captures inter-channel feature correlations by computing the Gram matrices of the feature maps, thereby modeling the statistical style characteristics of the image. By minimizing the differences between the style matrices of the reconstructed and original images across multiple feature levels, the model can more effectively restore the input image’s texture patterns, color distribution, and overall visual style, significantly improving the visual naturalness of the reconstruction results.

### Architectural configuration

The bottom encoder (Enc$_b$) uses two strided convolutional layers (4$\times$4 kernel, stride 2, with 64 and 128 output channels), followed by a 3$\times$3 convolution and two residual blocks (128 channels, 32-channel bottleneck). The top encoder (Enc$_t$) applies a further downsampling convolution (4$\times$4, stride 2, 128$\rightarrow$64 channels), a 3$\times$3 convolution to 128 channels, and two residual blocks of identical configuration. Both top and bottom quantizers project features to a 64-dimensional embedding space via 1$\times$1 convolutions before vector quantization. The top decoder (Dec$_t$) reconstructs top-level quantized features through a 3$\times$3 convolution (64$\rightarrow$128), two residual blocks, and a transposed convolution (4$\times$4, stride 2) that upsamples to the spatial resolution of the bottom-level features.

In the DPDF-Decoder, the concatenated top and bottom features (totaling 128 channels) are fed into two parallel branches, each starting with a 3$\times$3 convolution (128$\rightarrow$128), batch normalization, and ReLU. The low-frequency branch processes features through two residual blocks and applies progressive bilinear upsampling (two 2$\times$ operations) for a total 4$\times$ resolution increase. The high-frequency branch employs one residual block followed by two PixelShuffle operations, each performing 2$\times$ upsampling via channel-to-spatial rearrangement. The dual-path outputs are concatenated (256 channels) and fused through a 1$\times$1 convolution (256$\rightarrow$128), batch normalization, ReLU, one residual block, and a final 3$\times$3 convolution producing the RGB output. The total parameter count of HiRes-VQ is 3.21M.

**Fig. 4:**
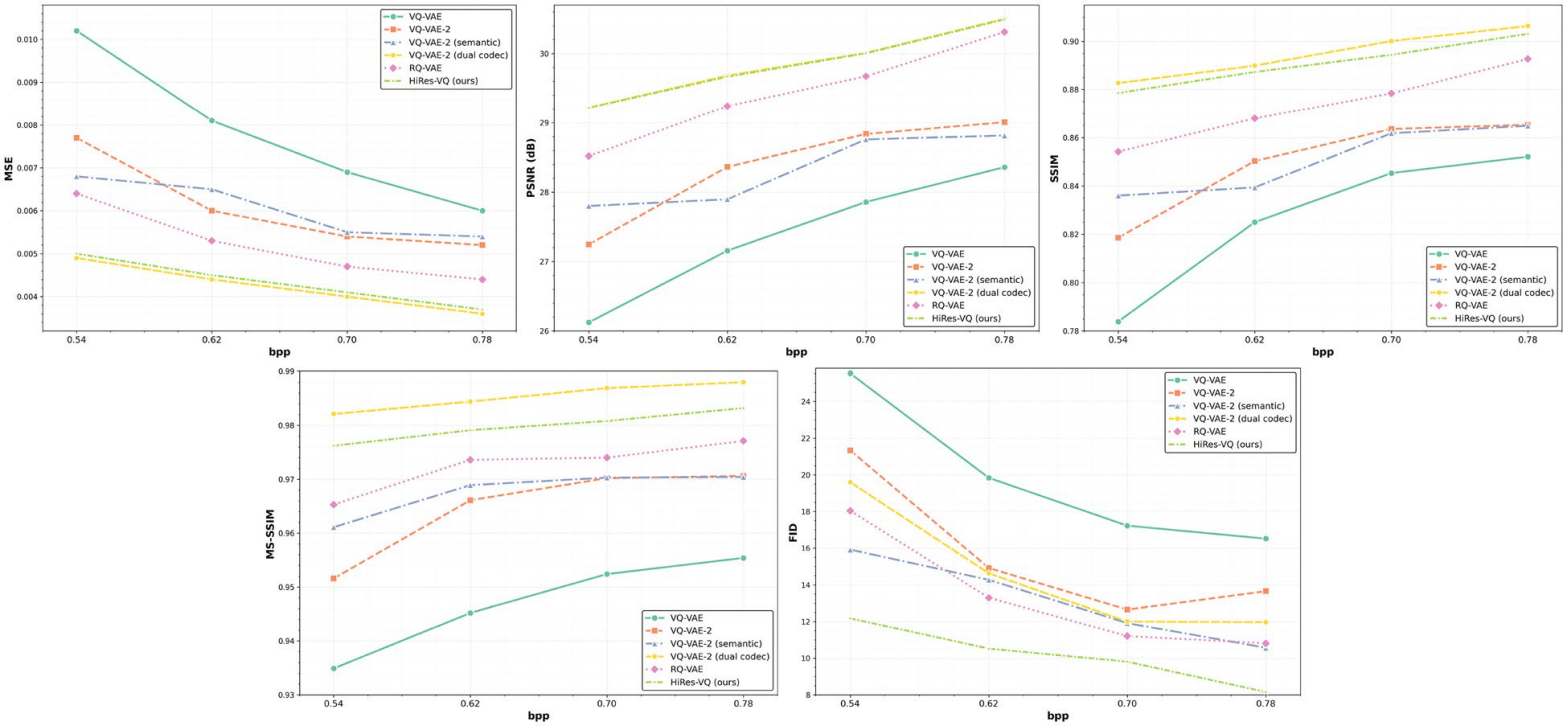
indexes on ImageNet-256.

## Experiments

### Experimental setup

- *Datasets. *We conduct comprehensive experimental evaluations on two large-scale image datasets: FFHQ-256 (containing 70,000 high-quality facial images) and ImageNet-256 (100,000 natural scene images randomly selected from the ImageNet dataset). All images are preprocessed to a resolution of 256 $\times$ 256 and divided into training and validation sets following standard protocols.
- *Evaluation metrics. *We employ a multi-dimensional quality assessment system: Mean Squared Error (MSE) and Peak Signal-to-Noise Ratio (PSNR) measure pixel-level reconstruction accuracy; Structural Similarity Index (SSIM) and Multi-Scale Structural Similarity Index (MS-SSIM) evaluate structural fidelity; Fréchet Inception Distance (FID) quantifies the perceptual similarity between the distributions of generated and real images. All metrics are calculated as averages over the same test set.
- *Training configuration. *All models are trained using the Adam optimizer with an initial learning rate of $3\times 10^{-4}$ and a cosine annealing scheduler. The batch size is set to 32. Training is performed for 560 epochs on a single NVIDIA RTX 4090 GPU. All comparative experiments are conducted under identical hardware and software environments to ensure fairness.
- *Implementation details. *The four bit rates (0.54, 0.62, 0.70, and 0.78 bpp) are achieved by varying the codebook size $K \in \{128, 256, 512, 1024\}$ while keeping the embedding dimension fixed at 64 for both top and bottom quantizers. The loss weights are configured as $\lambda _{\text {mse}}=1.0$, $\lambda _{\text {commit}}=0.25$, $\lambda _{\text {perc}}=0.1$, and $\lambda _{\text {style}}=10^{-10}$. The perceptual loss employs a pretrained VGG-16 network with features extracted from the conv1_1, conv2_1, conv3_1, conv4_1, and conv5_1 layers (after ReLU), weighted equally ($\alpha _l=1.0$ for all layers). The perceptual and style weights follow a dynamic schedule decaying by a factor of $1/(\text {epoch}+1)$.

### Comparison of reconstruction quality with VQVAE series models

- *Analysis of quantitative results. *To evaluate model performance across compression ratios, we conducted experiments at four bit rates (0.54, 0.62, 0.70, and 0.78 bpp) based on HiRes-VQ codebook sizes. All baseline models maintained a similar parameter count (3.2M) to ensure a fair comparison.

We compare our proposed model with three baseline methods (VQ-VAE, VQ-VAE-2, and RQ-VAE, where the latter applies RQ-VAE’s residual quantizer module to VQ-VAE-2 to maintain a comparable parameter count) and two ablation variants—VQ-VAE-2 augmented with only the dual-path decoder (VQ-VAE-2 + dual dec), and VQ-VAE-2 augmented with only the perceptual and style losses (VQ-VAE-2 + loss). All models maintain a similar parameter budget (3.2M). The results demonstrate that our full model significantly outperforms all other configurations across all evaluated metrics at every bit rate.

MSE, the most direct pixel-wise error metric, shows that lower values indicate higher reconstruction accuracy. As shown in the upper-left subplot of Fig. 2, all curves decline as the bit-rate increases, but the curve for HiRes-VQ consistently remains at the bottom, maintaining a clear gap from all comparison and ablation configurations. This advantage is particularly pronounced in the lower bit-rate range, demonstrating that HiRes-VQ utilizes limited bits more effectively to enhance reconstruction accuracy under constrained transmission conditions. When the bit-rate reaches 0.78 bpp, the MSE of HiRes-VQ approaches 0.0015, whereas the best-performing baseline, RQ-VAE, remains above 0.0020. This gap intuitively confirms the superiority of our method in pixel-level error control.

PSNR is a core objective quality metric in pixel-level image compression, with higher values representing lower reconstruction distortion. As depicted in the upper-right subplot of Fig. 2, the PSNR curve for HiRes-VQ consistently stays above those of the other models, and this advantage remains stable as the bit-rate increases. At 0.54 bpp, the PSNR of HiRes-VQ already surpasses the levels achieved by VQ-VAE and VQ-VAE-2 at 0.78 bpp, demonstrating that HiRes-VQ can achieve reconstruction quality comparable to high-bit-rate baselines at a lower bit-rate. Furthermore, the slope of the HiRes-VQ curve remains relatively smooth across all bit-rate segments without significant saturation, suggesting its potential for further improvement at even higher bit-rates.

SSIM and MS-SSIM assess reconstruction fidelity from the perspective of structural similarity, aligning more closely with human perception; their respective curves are shown in the lower part of Fig. 2. The SSIM curve for HiRes-VQ reaches 0.925 at 0.54 bpp, a value that other models only approach at higher bit-rates (0.70 bpp and above). For the MS-SSIM metric, HiRes-VQ maintains a similarly significant advantage, which is even more pronounced in the lower bit-rate range. Additionally, both the SSIM and MS-SSIM curves for HiRes-VQ are positioned at a high level and exhibit a relatively gentle slope; the increase from 0.54 bpp to 0.78 bpp is smaller than that of the comparison models. This indicates that HiRes-VQ effectively captures the structural information of images even at the lowest bit-rate, with subsequent increases in bit-rate primarily contributing to the refinement of textural details rather than structural correction.

**Fig. 5:**
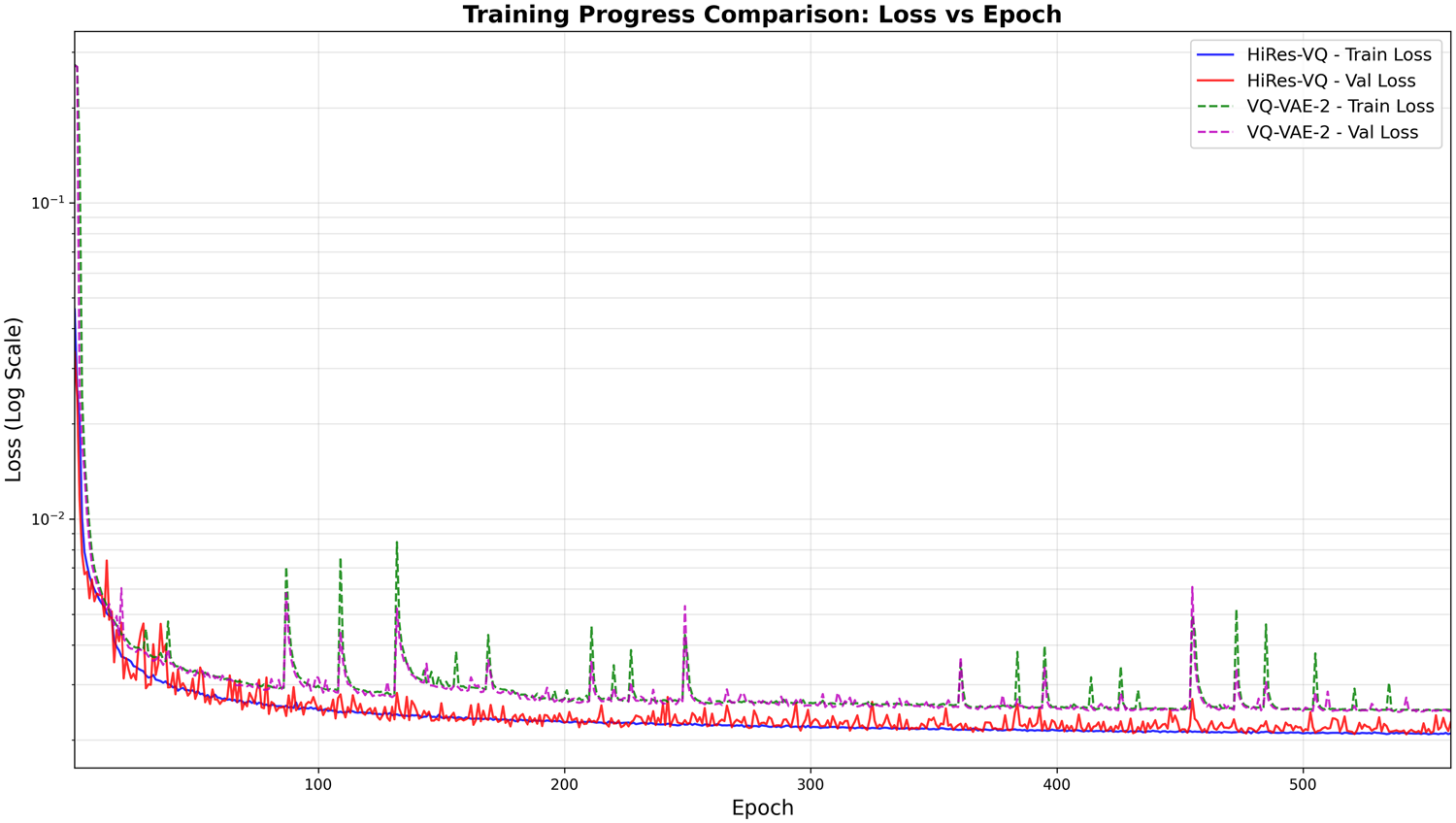
Training progress comparison on FFHQ-256.

Figure 3 illustrates the FID curves across different bit-rates for all compared configurations. Overall, the FID values for all models decrease as the bit-rate increases, indicating that higher bit-rates assist the models in recovering richer semantic details. There is a significant and consistent ranking in the FID scores: the FID curve for HiRes-VQ consistently stays at the bottom, while VQ-VAE is at the top; the ablation variants VQ-VAE-2 + loss and VQ-VAE-2 + dual dec fall between VQ-VAE-2 and HiRes-VQ, with the perceptual-loss variant outperforming the decoder-only variant in semantic quality. This ranking aligns with the pixel-level metrics, further confirming the hierarchical differences among the models in their ability to preserve semantic information. At the lowest bit-rate of 0.54 bpp, the FID of HiRes-VQ reaches 26.32, which is 40.9% lower than that of VQ-VAE (44.52), 26.1% lower than VQ-VAE-2 (35.61), and 18.1% lower than RQ-VAE (32.12). This significant advantage demonstrates that under the same low bit-rate constraint, HiRes-VQ can generate reconstructed images that are semantically far superior to the baselines, appearing more realistic and closer to the original data distribution, while the comparison models exhibit varying degrees of semantic blurriness or structural distortion. As the bit-rate increases to 0.78 bpp, the FID of HiRes-VQ further decreases to 21.02, still significantly ahead over RQ-VAE (23.64), VQ-VAE-2 (27.85), and VQ-VAE (32.00). Notably, the FID of HiRes-VQ at 0.54 bpp (26.32) even outperforms those of VQ-VAE and VQ-VAE-2 at 0.78 bpp. This cross-bitrate comparison directly demonstrates that our model can achieve semantic preservation superior to higher-bitrate baseline models using only about 70% of the bit budget, fully validating its ability to prioritize the retention of semantic information within limited bit-rate constraints.

To validate the generalization capability of the HiRes-VQ model across different image domains, we repeated the same experiment conducted on FFHQ using the ImageNet256 dataset, with results shown in Fig. 4.

Regarding the overall trend, the experimental findings on ImageNet are highly consistent with those on FFHQ. HiRes-VQ maintains a comprehensive lead across all pixel-level and semantic-level fidelity metrics, demonstrating the excellent generalization performance of our model across different image domains and once again confirming the effectiveness and robustness of our proposed method.

To further elucidate the individual contribution of each key design component, the ablation variants plotted in Figs. 2, 3 and 4 allow us to isolate the effects of the dual-path decoder and the perceptual alignment loss. Starting from the VQ-VAE-2 baseline, VQ-VAE-2 + dual dec replaces the original decoder with our frequency-decoupled dual-path decoder while retaining only MSE and commitment losses, and VQ-VAE-2 + loss augments VQ-VAE-2 with the perceptual and style losses while keeping the decoder unchanged. The results reveal a pronounced functional division between the two components.

On the FFHQ-256 dataset at 0.54 bpp, VQ-VAE-2 + dual dec substantially improved pixel-level metrics over the VQ-VAE-2 baseline: SSIM increased by 3.2% (0.8819 $\rightarrow$ 0.9103) and MS-SSIM rose from 0.9749 to 0.9854, whereas FID saw only a modest reduction of 4.8% (35.61 $\rightarrow$ 33.90). In stark contrast, VQ-VAE-2 + loss left pixel-level metrics largely unchanged: SSIM edged up by merely 0.1% (0.8819 $\rightarrow$ 0.8828) and MS-SSIM marginally declined from 0.9749 to 0.9737, yet delivered a far more substantial FID reduction of 14.3% (35.61 $\rightarrow$ 30.53). The full HiRes-VQ, by integrating both components, achieved the best of both worlds: SSIM of 0.9135 and FID of 28.69 (a 19.4% reduction over VQ-VAE-2), demonstrating significant semantic-level gains without any sacrifice in pixel-level fidelity. This pattern held consistently across bit rates: at 0.78 bpp, VQ-VAE-2 + dual dec improved SSIM to 0.9343 but reduced FID only to 24.19, while VQ-VAE-2 + loss retained an SSIM close to the baseline (0.9213 vs. 0.9178) yet reduced FID to 22.90; HiRes-VQ achieved the best values on both metrics (SSIM 0.9342, FID 22.38).

The same contrasting pattern emerged on the more diverse ImageNet-256 dataset, where the pixel-level degradation of the loss-only variant was occasionally more visible. At 0.62 bpp, for example, VQ-VAE-2 + loss saw its PSNR drop from 28.36 (VQ-VAE-2) to 27.90 and its SSIM decline from 0.8503 to 0.8394, while still achieving a substantial FID improvement (14.92 $\rightarrow$ 14.27). Meanwhile, VQ-VAE-2 + dual dec at the same bit rate improved PSNR from 28.36 to 29.68 and SSIM from 0.8503 to 0.8899, with a comparatively smaller FID gain (14.92 $\rightarrow$ 14.63). At 0.54 bpp, VQ-VAE-2 + dual dec improved SSIM by 7.8% (0.8186 $\rightarrow$ 0.8827) with only an 8.1% FID reduction (21.34 $\rightarrow$ 19.60), whereas VQ-VAE-2 + loss reduced FID by 25.4% (21.34 $\rightarrow$ 15.92) with only a 2.1% SSIM gain. The full HiRes-VQ achieved SSIM of 0.8786 and FID of 12.18 at 0.54 bpp—a 42.9% FID reduction over VQ-VAE-2 that exceeds the sum of the individual contributions, indicative of a synergistic interaction. At 0.78 bpp, HiRes-VQ reduced FID from 13.66 (VQ-VAE-2) to 8.16, a 40.3% improvement, while maintaining competitive pixel-level accuracy (SSIM 0.9031 vs. 0.9063 for VQ-VAE-2 + dual dec).

These results conclusively demonstrate that the two components serve distinct and complementary roles: the frequency-decoupled dual-path decoder is the primary driver of pixel-level fidelity and structural accuracy, with limited impact on semantic quality; the multi-scale perceptual alignment loss is the primary driver of semantic-level perceptual quality, yet when applied in isolation it provides negligible pixel-level benefit and can even slightly degrade structural metrics. Only by combining both components does HiRes-VQ achieve a substantial and simultaneous improvement across both pixel-level and semantic-level metrics, effectively resolving the quality-efficiency dilemma without resorting to adversarial training or increased model complexity.

**Fig. 6:**
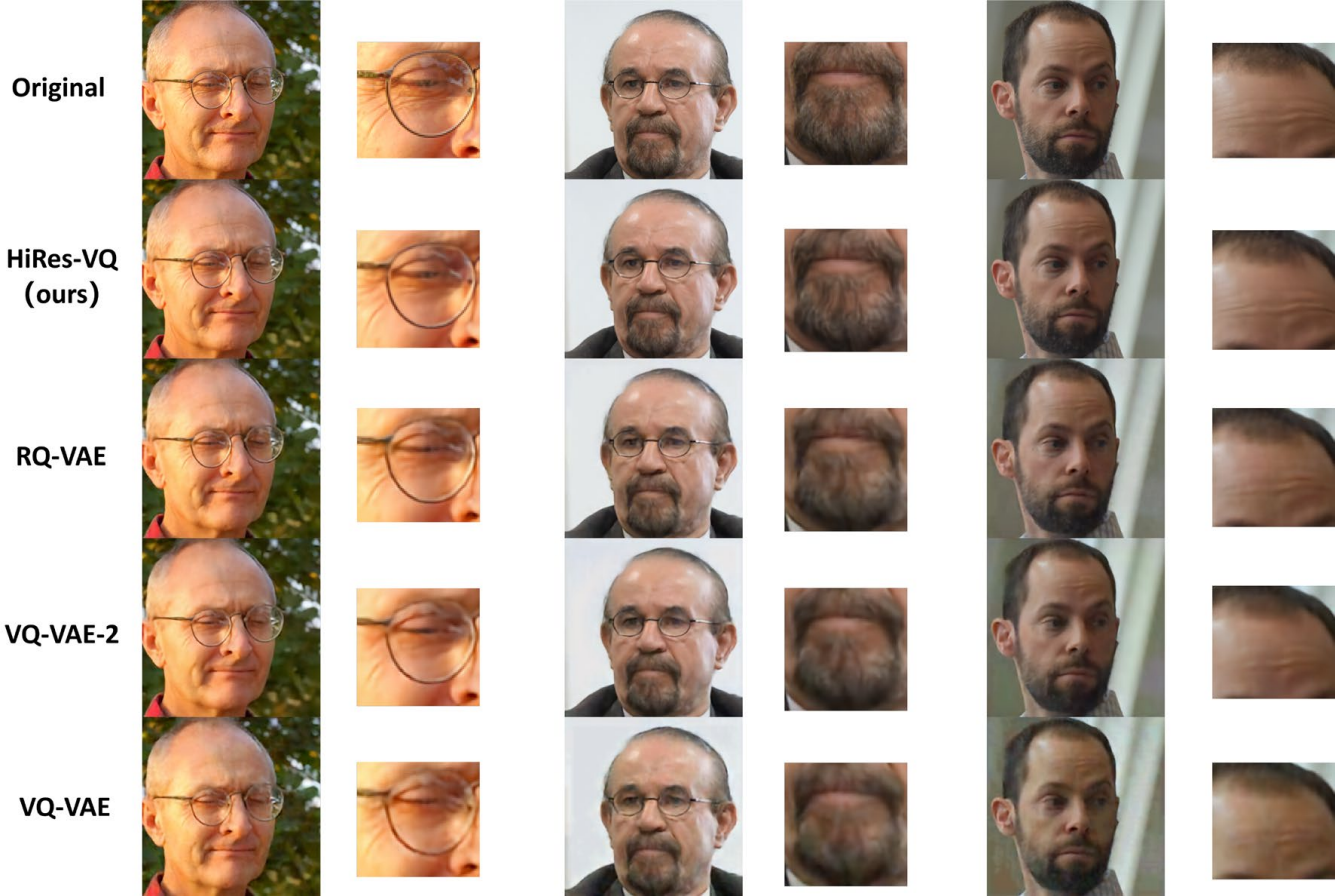
Reconstruction comparison on FFHQ-256.

- *Training stability and rate of convergence.* Figure 5 provides a comparison of the training dynamics between VQ-VAE-2 and our proposed model (HiRes-VQ), aiming to illustrate the differences in learning efficiency, generalization capability, and optimization difficulty across the different model architectures.

In terms of convergence speed, the training loss of HiRes-VQ was 0.0460 after the first epoch and rapidly decreased to 0.0041 after only 17 epochs, a reduction of 91.1%. Subsequently, the loss continued to decline smoothly, stabilizing around 0.0024 by the 80th epoch, and eventually reaching a minimum of approximately 0.0021 at the 521 st epoch. In contrast, the convergence process of VQ-VAE-2 was notably slower: its initial training loss was as high as 0.2732, and it took about 40 epochs to fall below 0.0035. Moreover, its loss was still fluctuating around 0.0030 even after 100 epochs, requiring approximately 2.5 times the number of epochs needed by HiRes-VQ to converge. This comparison strongly indicates that the hierarchical feature extraction and dual-path decoder mechanism adopted by HiRes-VQ effectively accelerate the loss reduction process, enabling the model to capture the core semantic structure of images within a very short training period.

Regarding training stability, the training loss curve of HiRes-VR is almost entirely smooth, and its validation loss exhibits minimal fluctuation, without any noticeable loss rebounds or spikes. In contrast, the training process of VQ-VAE-2 frequently shows drastic loss fluctuations. At multiple epochs, both its training and validation losses suddenly spike upwards before slowly decreasing again, forming conspicuous ”spikes”. This recurrent instability not only prolongs the effective convergence time but also reflects that the standard VQ-VAE-2 is susceptible to interference from gradient anomalies or conflicts in quantizer updates during training. The ability of HiRes-VQ to maintain highly stable training dynamics is attributed to the smooth constraint imposed on the feature space by its multi-level perceptual alignment loss, as well as the stable gradient pathways provided by the residual block design within its decoder. These design elements collectively suppress factors that cause oscillations during training, allowing the model to smoothly approach the optimal solution.

- *Visual comparison.* Figure 6 presents the visual comparison results on the FFHQ-256 dataset. Under low bit-rate configurations, the reconstruction results from the original VQ-VAE model exhibit significant blurring and loss of detail, particularly in high-frequency regions. While VQ-VAE-2 and RQ-VAE show some improvement, issues such as texture smoothing and structural blurring persist.

In contrast, the reconstruction results from our model preserve clear structures while recovering a greater amount of high-frequency detail. Details such as skin texture, hair strands, and eye highlights in FFHQ facial images are better retained. For the more complex images in ImageNet, our model can more accurately reconstruct fine-grained textures, such as animal fur, leaf venation, and the surface textures of building materials.

**Fig. 7:**
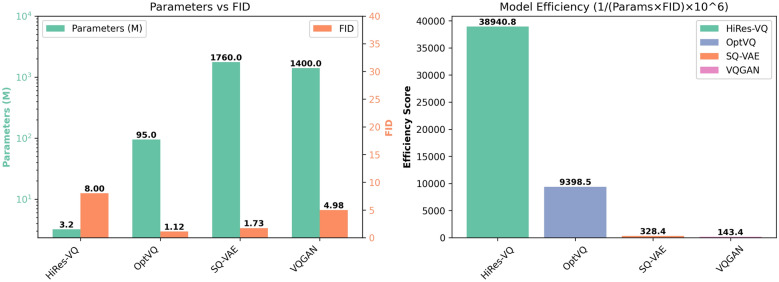
Comprehensive performance comparison with advanced models.

- *Comprehensive performance comparison with advanced models.* Figure 7 presents a comprehensive performance comparison between our model and several advanced models on the ImageNet-256 dataset, including SQ-VAE, VQGAN, and OptVQ.

9 $$\begin{aligned} E_{\text {model}}=\frac{10^6}{N_{\text {param}} \times \text {FID}}. \end{aligned}$$

From the perspective of the model quality-efficiency evaluation metric (Eq. 9), our model significantly outperforms the others. In terms of training efficiency, our model also demonstrates substantial advantages: it has only 3.21M parameters, which is just 4% of OptVQ’s (90M)^22^ and 0.2% of VQGAN’s (1.4B). Compared to VQGAN, our training time is reduced from 14 days to 3 days. This improvement in efficiency primarily stems from our lightweight model design and stable training strategy. This advantage makes our model more suitable for deployment in environments with limited training duration and computational resources.

- *Influence of image resize on training outcome.* To investigate the impact of input image resolution on model training effectiveness and efficiency, we compared the reconstruction performance and training time consumption on the FFHQ and ImageNet datasets under two settings: using 128$\times$128 and 256$\times$256 as the resizing dimensions for input images originally at 256$\times$256 resolution. All experiments were conducted with identical model architectures and training hyperparameters. Table 1 summarizes the MSE, PSNR, SSIM, MS-SSIM, and FID metrics corresponding to different bit-rates under both settings, as well as the training time consumption per epoch.

The results from the FFHQ dataset show that, at the same bit-rate, the models using 256$\times$256 input resolution outperformed those using 128$\times$128 across all evaluation metrics. As the bit-rate increased, the performance gap between the two settings narrowed relatively. A similar trend was observed on the ImageNet dataset. This phenomenon indicates that higher input resolution provides the model with richer spatial detail information, enabling the encoder to extract finer texture and edge features, thereby restoring visual content closer to the original image during the quantization and reconstruction process.

It is worth noting that although the performance of the 128$\times$128 setting was slightly inferior to that of the 256$\times$256 setting, the degree of degradation was relatively limited. At the same time, the training efficiency improvement brought by the 128$\times$128 resolution was significant: on the FFHQ dataset, the training time per epoch was reduced from 8 minutes (256$\times$256) to 2.5 minutes (128$\times$128), compressing the time to 31% of the original; on the ImageNet dataset, it decreased from 14 minutes to 4.5 minutes, compressing it to 32%. This implies that in scenarios with limited computational resources or where rapid iteration is required, using a 128$\times$128 input can substantially shorten training time at the cost of only a minimal sacrifice in reconstruction quality, making it more suitable for practical applications in resource-constrained environments.

**Table 1:** Performance comparison across different input resolutions and datasets.

Dataset	Size	Time	bpp	MSE$\downarrow$	PSNR$\uparrow$	SSIM$\uparrow$	MS-SSIM$\uparrow$	FID$\downarrow$
FFHQ	128	2.5 min	0.54	0.0024	32.23	0.9135	0.9853	28.6863
			0.62	0.0021	32.92	0.9261	0.9884	23.9955
			0.70	0.0019	33.31	0.9306	0.9892	23.5011
			0.78	0.00017	33.64	0.9342	0.9903	22.3840
FFHQ	256	8 min	0.54	0.0020	32.83	0.9247	0.9875	26.3188
			0.62	0.0019	33.28	0.9301	0.9890	22.8931
			0.70	0.0017	33.61	0.9340	0.9904	21.9957
			0.78	0.0016	33.95	0.9402	0.9913	21.0235
ImageNet	128	4.5 min	0.54	0.0050	29.22	0.8786	0.9762	12.1797
			0.62	0.0045	29.67	0.8872	0.9791	10.5228
			0.70	0.0041	30.00	0.8943	0.9808	9.8088
			0.78	0.0037	30.49	0.9031	0.9832	8.1558
ImageNet	256	14 min	0.54	0.0047	29.62	0.8863	0.9785	11.3851
			0.62	0.0042	30.07	0.8930	0.9803	10.0471
			0.70	0.0039	30.31	0.9028	0.9812	9.2830
			0.78	0.0036	30.75	0.9107	0.9836	8.0073

Lower MSE and FID, higher PSNR, SSIM, and MS-SSIM indicate better reconstruction quality.

## Conclusion

This study tackles the quality-efficiency trade-off in low-bit-rate image compression. We propose a lightweight, high-quality model that balances complexity, speed, and fidelity. By enhancing VQ-VAE-2 with a novel super-resolution codec, it decouples low-frequency structure and high-frequency detail reconstruction, improving visual quality within a compact design.A multi-level perceptual loss, combining pixel accuracy, semantic feature matching, and style constraints, guides the model to learn human vision-aligned representations.

Experiments on FFHQ-256 and ImageNet-256 show our model significantly outperforms traditional VQ-VAE variants in metrics like FID and MS-SSIM, while maintaining comparable parameters and speed. Against high-complexity models like VQGAN, it achieves leading quality-efficiency metrics with drastically lower computational cost, validating its effective balance of quality and efficiency.

## Limitations and future work

Despite the promising results, several limitations of the current work warrant discussion and motivate directions for future investigation.

The compression capability of HiRes-VQ still fundamentally relies on the vector quantization mechanism inherited from VQ-VAE, which achieves compression by mapping continuous encoder features to discrete codebook indices. However, the resulting index map retains substantial spatial and semantic redundancy due to the non-uniform distribution of codebook assignments across spatial locations. Unlike modern learned compression methods that employ context-adaptive entropy models to further exploit this redundancy for bit-rate reduction, the current framework applies no additional entropy coding on top of the quantized indices. This leaves room for further compression gains through techniques such as autoregressive entropy models over the latent code grid or spatially-aware bit allocation strategies. Future work could explore integrating a learned entropy model into the HiRes-VQ pipeline to more effectively exploit the remaining statistical redundancy in the discrete latent representation.

Moreover, while the current evaluation covers a range of low bit rates (0.54–0.78 bpp), the model’s behavior at ultra-low bit rates (e.g. 0.1 bpp) remains unverified. At such extreme compression ratios, the limited capacity of the discrete latent representation may pose fundamental challenges for preserving both structural coherence and perceptual fidelity. The trade-off between the bit budget allocated to low-frequency structure recovery versus high-frequency texture reconstruction may shift substantially in this regime, and the relative contributions of the dual-path decoder and perceptual loss observed at moderate bit rates may not directly extrapolate. Systematic investigation of HiRes-VQ under ultra-low-bit-rate conditions, potentially coupled with more aggressive dimensionality reduction in the encoder or adaptive bit allocation between the two decoder branches, constitutes an important direction for future work.

## Data Availability

The dataset FFHQ-256 analysed during the current study is available from https://github.com/NVlabs/ffhq-dataset\\ The dataset ImageNet-256 analysed during the current study is available from https://image-net.org/challenges/LSVRC/index.php All the human face images appear in the manuscript were extracted from the publicly available dataset FFHQ.
